# Tetracycline-resistant *Neisseria gonorrhoeae* global estimates—impacts on doxycycline post-exposure prophylaxis implementation and monitoring: a systematic review

**DOI:** 10.1093/jacamr/dlaf120

**Published:** 2025-07-09

**Authors:** Kim Do, Magnus Unemo, Chris Kenyon, Jane S Hocking, Fabian Yuh Shiong Kong

**Affiliations:** School of Medicine, The University of Notre Dame, Sydney, Australia; WHO Collaborating Centre for Gonorrhoea and Other STIs, National Reference Laboratory for STIs, Department of Laboratory Medicine, Örebro University, Örebro, Sweden; Institute for Global Health, University College London, London, UK; HIV/STI Unit, Institute of Tropical Medicine, Antwerp, Belgium; Division of Infectious Diseases and HIV Medicine, University of Cape Town, Cape Town, South Africa; Centre for Epidemiology and Biostatistics, Melbourne School of Population and Global Health, University of Melbourne, Melbourne, Australia; Centre for Epidemiology and Biostatistics, Melbourne School of Population and Global Health, University of Melbourne, Melbourne, Australia

## Abstract

**Objectives:**

Doxycycline post-exposure prophylaxis (doxyPEP) can reduce incident sexually transmitted infections including gonorrhoea for MSM and transgender women. Its effectiveness depends on the level of tetracycline resistance in *Neisseria gonorrhoeae*, which varies by country. Countries implementing doxyPEP should have robust antimicrobial resistance (AMR) surveillance using standardized, quality-assured methods. This systematic review estimates the proportion of tetracycline-resistant *N. gonorrhoeae* isolates by country/region and describes the contribution of sex and infection site to these estimates.

**Methods:**

We searched bibliographic databases (1 January 2000 to 26 August 2024) for English-language studies reporting tetracycline MIC with a sample size of >10 isolates. Data on country, year, sex, sexual orientation and infection site were collected. Countries were grouped into seven World Bank regions. Tetracycline resistance (MIC > 1 mg/L) was reported by country, region and time period (2010–23 versus 1996–2009).

**Results:**

Sixty-seven included studies from 51 countries studying 80 645 isolates (91% from 2010–23) were analysed. Overall median tetracycline resistance was 54.2% (range 4.0%–100.0%). Highest resistance occurred in East Asia and Pacific (82.1%, 18%–100%) and sub-Saharan Africa (81.6%, 44%–100%), and lowest in North America (26.5%, 4%–78%). Only 16% (11/67) of studies reported MSM, 18% (12/67) included oropharyngeal isolates and 9% (6/67) included women. Resistance increased by 3–4-fold in South Asia [relative risk (RR) 3.8] and North America (RR 4.1) over time.

**Conclusions:**

High and rising tetracycline resistance limits doxyPEP’s potential to prevent gonorrhoea. More data are needed from MSM, women and oropharyngeal sites to understand AMR trends and transmission dynamics between MSM and women.

## Introduction

Gonorrhoea is the second leading reported sexually transmitted infection (STI) globally, with 82.4 million estimated cases in 2020.^1^ Gonorrhoea infects both urogenital and extragenital (oropharyngeal and anorectal) sites and while extragenital infections are mostly asymptomatic,^2^ genital infections in men are symptomatic in 90% of cases, while about 40% of genital infections in women may present with non-specific symptoms.^2^

In particular, MSM are at a disproportionately higher risk of gonorrhoea. Recent surveillance data have shown significant increase in *Neisseria gonorrhoeae* infections in MSM populations.^3–5^ To mitigate these rates, the use of doxycycline as post-exposure prophylaxis (doxyPEP) has been trialled to prevent STIs, including *N. gonorrhoeae*, in the USA^6^ and in France^7,8^ among MSM and transgender women (TGW). These doxyPEP trials reported significant incidence reductions for chlamydia or syphilis by approximately 80%, and gonorrhoea by 55% in the USA^6^ and 33% in France.^8^ The differences in *N. gonorrhoeae* efficacy between the USA and France was likely affected by the lower levels of tetracycline resistance in the USA (20%–30%)^9^ compared with France (65%)^10^ at the time of the trials. In addition, participants in the USA were permitted to ingest more doses of doxyPEP than in France (seven versus three doses a week). However, incident gonorrhoea cases significantly increased (1.8% per month) in San Francisco, CA, USA during the first year after doxy-PEP was rolled out.^11^ A trial in *cis*-women in Kenya found doxyPEP was not effective against STIs, likely due to non-compliance, but for *N. gonorrhoeae* it was not effective as all isolates were resistant to doxycycline.^12^

Important consideration in implementing doxyPEP includes ensuring doxyPEP remains effective and does not contribute to the induction or selection of antimicrobial resistance (AMR) in STI agents such as *N. gonorrhoeae* and in non-STI agents or commensal bacterial species such as *Staphylococcus aureus*. While the numbers of *N. gonorrhoeae* isolates providing AMR data were low in both the USA (*n* = 68)^6^ and the French DoxyVAC (*n* = 78)^8^ study, both reported greater numbers of high-level tetracycline resistance at follow-up in the doxyPEP arm compared with standard of care. This resistance is particularly important for oropharyngeal infections, where treatment failure occurs more frequently,^13^ particularly among MSM, and where AMR is mostly generated.^2^ Of concern, lower clearance of oral *N. gonorrhoeae* was observed in both doxyPEP studies.^6,7^ Additionally in the USA study,^6^ there was a significant increase in doxycycline resistance in *S. aureus* between baseline and the 12 month visit (4% to 12%, *P* < 0.05). There was also an increase in doxycycline resistance in commensal *Neisseria* spp. in the oropharynx in the doxyPEP arm versus the control arm (70% versus 45%, *P* < 0.05). Lastly, the doxyPEP arm acquired resistance in *N. gonorrhoeae* to other classes of antibiotics such as azithromycin and ciprofloxacin that was not seen in the control arm. In the recent USA trial, doxyPEP use increased doxycycline-resistant *S. aureus* by nearly 4-fold (HR 3.89, 1.42–10.68, *P* = 0.0044).^14^ Lastly, in the DoxyVAC trial, increases in gonococcal cefixime MIC was also observed.^8^ Given these concerns, doxyPEP guidelines have called for ongoing surveillance, including of AMR, to be able to monitor tetracycline resistance in STIs, especially for *N. gonorrhoeae*, and for non-STIs such as human commensals.^15,16^

Therefore, in implementing doxyPEP in any country, understanding and monitoring background *N. gonorrhoeae* tetracycline resistance is an important consideration for *N. gonorrhoeae* control and will inform the need to inform users regarding its effectiveness against *N. gonorrhoeae* and continued emphasis on screening. This information is critical as countries around the world begin to recommend doxyPEP, such as in the USA^15^ and Australia,^17^ while other countries such as England,^18^ Germany^19^ and the EU^16,20^ take a more cautious approach.

Currently, there is a lack of reviews evaluating the extent of tetracycline resistance in *N. gonorrhoeae* globally. This study aims to undertake a systematic review of tetracycline resistance in *N. gonorrhoeae* and provide a narrative of the capacity of the current surveillance systems to collect adequate information (e.g. samples from MSM and from the oropharynx) to inform global policy on the utility of doxyPEP as a tool for *N. gonorrhoeae* control and for the ongoing monitoring of *N. gonorrhoeae* AMR.

## Materials and methods

This study protocol was registered on PROSPERO (CRD42023407932) and the results were reported according to PRISMA^21^ and Synthesis without meta-analysis (SWiM) guidelines;^22^ see Tables S1 and S2 (available as Supplementary data at *JAC-AMR* Online), respectively.

### Search strategy

We searched Ovid MEDLINE, Ovid Embase and PubMed from 1 January 2000 to 26 August 2024. The search terms and their associated MeSH terms used for the search were (gonorrhea OR gonorrhoea OR *Neisseria gonorrhoeae*) AND (antibiotic resistance OR drug resistance, microbial OR antimicrobial resistance) AND (tetracycline or doxycycline). Only papers published after the year 2000 were included. National surveillance reports and grey literature were also searched.

### Eligibility criteria

Studies were included if they reported tetracycline or doxycycline resistance in *N. gonorrhoeae* isolates from clinical samples, defined as an MIC above an established clinical resistance breakpoint at the time of publication, such as EUCAST (www.eucast.org/clinical_breakpoints) or CLSI (www.clsi.org) or the presence of the tetracycline resistance gene *tet*(M) without MIC values.

Tetracycline resistance was used as a proxy for doxycycline effectiveness as doxycycline MICs correlate with tetracycline MICs.^23^

Only studies published in English with at least 10 isolates were included.

### Data extraction and management

Microsoft Excel was used to collate the data. The following were extracted from each study: author, year *N. gonorrhoeae* isolates were collected, year published, number of *N. gonorrhoeae* isolates providing tetracycline/doxycycline resistance data, country, World Bank region, proportion of *N. gonorrhoeae* isolates from MSM population if explicitly reported, anatomical site of infection, tetracycline/doxycycline resistance MIC cut-off value, MIC_90_ of isolates, tetracycline/doxycycline resistance percentage, presence of tetracycline/doxycycline resistance genes and method of determining the MIC. The sex, sexual orientation, age and anatomical site of the infection of *N. gonorrhoeae* isolates was extracted if available. Tetracycline/doxycycline resistance data were extracted from the most recent time period if the study was over multiple years. Where MIC data were measured using more than one method, such as ETEST, other MIC gradient strip test or agar dilution, results from agar dilution was extracted.

Studies that only used disc diffusion were excluded as this is not a sufficiently reliable or recommended method for detection of all levels of tetracycline/doxycycline resistance. Papers that exclusively studied tetracycline/doxycycline high-level resistance, i.e. MIC ≥ 16 mg/L, were also excluded. For USA eGISP data, 2019^24^ data were used as isolates by three anatomical sites in MSM was provided and MSM is the focus of this review.

One author (K.D.) extracted data independently, which was then reviewed by a second author (F.Y.S.K.). Any incongruence in the data was reviewed by a third author (J.S.H.).

### Outcome

The primary outcome was the proportion of clinical *N. gonorrhoeae* isolates with tetracycline resistance—with the numerator as the number of resistant isolates (defined as an MIC above a clinical breakpoint MIC or the presence of *tet*(M) in the absence of an MIC) and the denominator being the total of number of *N. gonorrhoeae* isolates.

Secondary outcomes were investigated for tetracycline resistance by region, temporality, site of infection, sex and sexual orientation.

### Data analysis

The primary outcome was the percentage of tetracycline resistance in *N. gonorrhoeae* isolates. Summary estimates (mean, median and range of the reported average values) were reported. In the review, median represents the median average values. Sub-analyses by country, World Bank regions (https://datatopics.worldbank.org/sdgatlas/archive/2017/the-world-by-region.html), site of infection and sex/sexual orientation were undertaken to investigate resistance by region and patient characteristics, if available. Analysis by temporality across two 14-year periods (resistance between years 1996–2009 compared with 2010–23) was estimated by regions to report changes in resistance over time. Six studies included data overlapping these two time periods and the final allocated time period was selected on the basis of the year contributing the majority of isolates that were collected. As such, one study with samples from 2004–05 and 2008–11 was included in the 1996–2009 category,^25^ with the remainder of the studies included in the 2010–23 group.^26–30^

Tetracycline resistance was reported in this review as the resistance defined and reported by the study authors. If the MIC defining resistance was not stated, the MIC of the reference antimicrobial susceptibility testing guidelines referenced in their methods, in the year the samples were collected, were applied, i.e. >1.0 mg/L for both EUCAST (before 2023) and CLSI.

If no MIC data were available, the presence of the tetracycline resistance gene *tet*(M) in an isolate was taken as a proxy for tetracycline resistance and included in the numerator to estimate resistance proportion since resistance (MIC ≥ 2 mg/L) would be expected.^31^ Where a study published both tetracycline and doxycycline MICs, tetracycline result was used as doxycycline MICs correlate with tetracycline MICs.^23^ Only one study^32^ provided doxycycline MIC data.

For one study,^33^ which collected isolates from countries across two regions (UK, USA and Canada), the study results were allocated to North America as the majority (63%) of isolates were from the USA and Canada. For the EU/EEA surveillance data report,^34^ the EUCAST MIC was used to estimate overall and regional resistance rather than the CLSI MIC.

Meta-analyses were not undertaken as many included studies did not have disaggregated data (e.g. by sex or site of infection) or reported the proportion of resistance as an average.

### Assessment of bias and quality

Formal assessment for bias was not conducted because no exposure or intervention effects were being calculated, therefore the typical domains for quality assessment such as randomization, performance bias, detection bias and attrition bias are less relevant. Additionally, included studies were not amenable to quality assessment because: (i) they are surveillance reports, thereby not constituting the traditional definition of a study; or (ii) they are laboratory studies using highly specific scientific methods that are not amenable to the quality assessment. As these included studies were mainly observational in nature, the data of interest were less vulnerable to author conflicts of interest or systematic bias.

## Results

### Study selection

**Figure 1.**
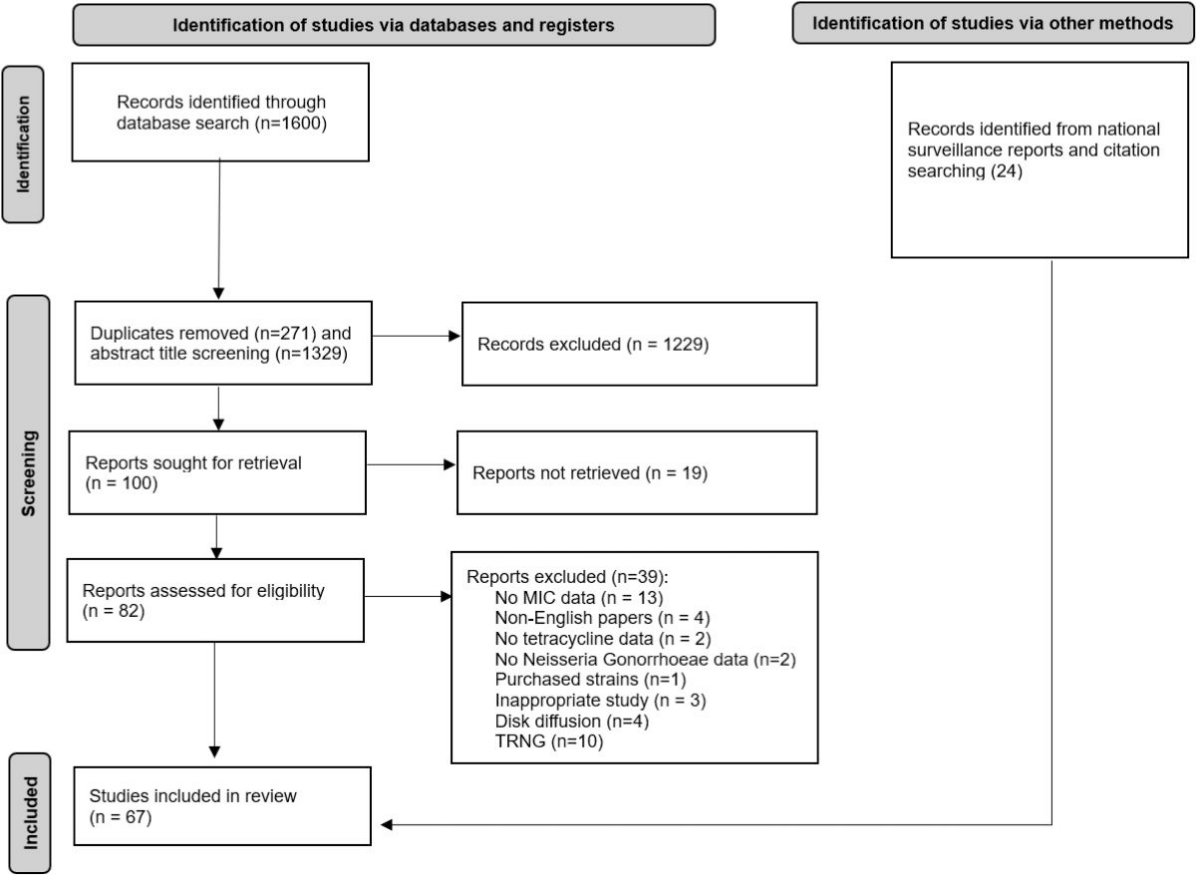
PRISMA flow chart for study selection and inclusion.

Overall, 100 studies were identified for retrieval and screening, of which 19 were excluded upon title and abstract screening, leaving 82 eligible for full-text screening (Figure 1). From these, 39 were excluded due to the following reasons: 13 had no MIC or resistance gene [*tet*(M)] data; 4 were not in the English language; 2 had no tetracycline data; 2 had no *N. gonorrhoeae* data; 1 tested only commercially available *N. gonorrhoeae* strains; 3 had inappropriate study design (replies to a publication, reported data from a publication); 4 used only the disc diffusion method; and 10 studied only high-level tetracycline resistance. Additionally, 24 studies were found from grey literature searching/citation searching or were national surveillance reports. Only the latest surveillance reports were included in the study, except for the US Gonococcal Isolate Surveillance Program (GISP), where we included both 2013 and 2023 reports as the 2013 study included data from MSM.^35^ Overall, data from 67 studies, 51 countries and 80 645 isolates—6917 between 1996 and 2009, and 73 728 between 2010 and 2023—were included.

### Study characteristics

#### Countries

Among included studies, one study each was conducted in Australia,^36^ Chile,^37^ Colombia,^37^ France,^38^ Japan,^39^ Korea,^40^ Madagascar,^41^ New Zealand,^42^ Pakistan,^43^ Poland,^44^ Portugal,^45^ Qatar,^46^ Switzerland,^27^ Thailand,^47^ Uruguay,^37^ Kenya^48^ or Zambia.^49^ Two studies each were from Argentina,^37,50^ Germany,^51,52^ Hungary,^53,54^ India,^29,55^ Indonesia,^32,56^ Malawi^57,58^ or Uganda.^58,59^

Three studies each were from Brazil,^26,60,61^ Cuba^37,62,63^ or Italy.^64–66^ Four studies were from Canada^33,67–69^ or England/Wales,^33,70–72^ five studies were from Russia^73–77^ or South Africa,^58,78–82^ seven were from the USA^24,33,35,83–86^ and nine studies were from China.^25,87–94^

Five studies included isolates from multiple countries. One study had isolates from Uganda, Malawi, South Africa, Kenya and Burkina Faso.^58^ The second study had isolates from Argentina, Bolivia, Chile, Colombia, Cuba, Uruguay and Venezuela.^37^ The third study included isolates from England, the USA and Canada.^33^ Lastly, one surveillance report from Europe (ECDC-funded Euro-GASP surveillance in EU/EEA) was included with isolates from Austria, Belgium, Bulgaria, Czechia, Estonia, France, Germany, Greece, Hungary, Ireland, Malta, Norway, The Netherlands, Poland, Portugal, Slovakia, Slovenia, Spain and Sweden.^34^

### Sex and sexual orientation

Regarding the sex of the study population, 10 (14.9%) studies^33,34,37,40,43,50,75,76,78,87^ did not specify the sex of participants, 1 (1.5%) study was conducted in women only,^56^ and in 5 (7.5%) studies women constituted 30%–58% of the study population.^32,41,47,59,69^ Fourteen (20.9%) studies were conducted only in men,^24,35,39,45,55,57,58,61,79,81,83,85,91,92^ with the remaining 35 studies (52.2%) including mostly (at least 73%) men. Eleven (16.4%) studies reported data on MSM also, who constituted 19%–100% of the study population.^32,35,38,45,53,60,66,70,83,85^

### Site of infection

All studies except 14 (20.9%)^25,33,37,40,52,63,68,70,71,75,76,86,87,92^ provided data on the proportion of isolates from urogenital sites (median 100% urogenital isolates, range 23.9%–100%). Twelve (17.9%) and 12 (17.9%) studies reported proportion of isolates from the anorectum^24,26,36,38,42,53,54,64,66,67,69,72^ (median 0%, 1.7%–69%) and oropharynx^24,27,36,42,44,53,54,64,66,67,69,72^ (median 0%, 1.2%–24.1%) site, respectively. No study except one^24^ provided site-specific MIC data for extragenital sites.

### Resistance by World Bank region

Overall, across all included studies, the median tetracycline resistance was 54.2% (range 4.0%–100.0%). The highest resistance levels were reported in East Asia and Pacific [*n* = 16, 82.1% (17.6%–100.0%)] and sub-Saharan Africa [*n* = 10, 81.6% (44.0%–100.0%)], while the lowest resistance levels were reported in North America [*n* = 10, 26.5% (4.0%–77.7%)] (Table 1). For other regions, median resistance ranged from 38.9% to 61.6%.

**Table 1.** Summary of tetracycline resistance in *N. gonorrhoeae* by country/region and patient characteristics (highest to lowest resistance)

Author, year published	Country	Year samples collected	No. isolates	Setting	% Male^b^	MSM data	Age (years)	Infection site	% Tetracycline resistance	Study resistance threshold MIC^c^ (≥mg/L unless otherwise stated)	MIC method
Sub-Saharan Africa^a^ (*n* = 10; 1733 isolates), median resistance 81.6% (range 44.0%–100%)											
Mabonga, 2019^59^	Uganda	2015	16	HIV outpatient service and sex worker clinic	43.8	No	>14 [median 24 (IQR 21–41.5)]	100% urogenital	100.0	≥2	Agar dilution
Kakooza, 2023^58^	Africa	Uganda (2016–20), Malawi (2017–19), South Africa (2015–19), Kenya (2013–18) and Burkina Faso (2018–19)	921	Sentinel surveillance sites including hospital, primary health care centres	100	Not provided	Uganda (16–79), Malawi (18–55), South Africa (18–46)	100% urogenital	94.2	Not provided—states per EUCAST (>1) or CLSI (≥2)	MIC gradient strip test (bioMérieux)
Rafetrarivony, 2024^41^	Madagascar	2014–20	24	Medical Laboratory	42	Not provided	Women median = 30, men median = 34	98.2% urogenital specimens plus also from sperm (*n* = 3), blood (*n* = 1), puncture fluid (*n* = 2) and a ‘not specified’ wound (*n* = 1)	87.0	Per CA-SFM (>1)	MIC gradient strip test (bioMérieux)
Kivata, 2020^48^	Kenya	2013–18	36	Clinics	Both	Not provided	Not provided	100% urogenital	86.1	>1	MIC gradient strip test (bioMérieux)
Brown, 2010^57^	Malawi	2007	100	STI clinic	100	Not provided	Not provided	100% urogenital	77.0	≥2	MIC gradient strip test (AB BIODISK), Agar dilution
Fayemiwo, 2011^79^	South Africa	2008	209	Not provided	100	Not provided	Not provided	100% urogenital	75.1	>1	MIC gradient strip test (AB BIODISK)
Peters, 2023^82^	South Africa	2022–23	100	Primary health care facilities	89	Not provided	18–50 (median 24)	100% urogenital	75.0	>1	MIC gradient strip test (bioMérieux)
Sarenje, 2022^49^	Zambia	2019–20	122	STI clinic in hospitals	73	Not provided	15–54	100% urogenital	68.9	≥2	MIC gradient strip test (bioMérieux)
De Jongh, 2007^81^	South Africa	2004–05	141	Primary healthcare and private clinics	100	Not provided	Not provided	100% urogenital	54.0	≥2	Agar dilution
Yakobi, 2023	South Africa	Not provided	64	Not provided	Not provided	No	Not provided	100% urogenital	44.0	>1	Disc diffusion and MIC gradient strip test (bioMérieux)
East Asia and Pacific (*n* = 16; 6304 isolates), median resistance 82.1% (range 17.6%–100%)											
Ieven, 2003^56^	Indonesia	1996	85	STI screening program	0 (100% female)	No	Not provided	100% urogenital	100.0	≥2	Agar dilution
Hananta, 2016^32^	Indonesia	2014	78	STI clinic and outreach venues	56.2	Yes (35.2% MSM)	>16	100% urogenital	98.7	>1	MIC gradient strip test (bioMérieux)
Liao, 2023^87^	China	2021	50	Surveillance—hospitals	Not provided	Not provided	Not provided	Not provided	98.0	>1	Agar dilution
Ye, 2002^94^	China	1996	352	STI clinic	Both, % not provided	Not provided	Not provided	100% urogenital	91.8	≥1	Agar dilution
Nokchan, 2022^47^	Thailand	2015–17	117	Hospital	45.3	Not provided	15 days–65 years (median 20)	93.1% urogenital: urethra (46.1%), cervix (29.1%), vagina (17.9%), eyes (4.3%) and other sites (2.6%). No site-specific MIC data	91.5	≥2	Agar dilution
Zhao, 2018^89^	China	2014	106	Hospital	73.1	Not provided	Not provided	100% urogenital	89.7	≥2	Agar dilution
Su, 2016^91^	China	2013	187	STI clinic	100	Not provided	Not provided	100% urogenital	87.2	≥2	Agar dilution
Gu, 2014^92^	China	2013	109	STI clinic	100	Not provided	Not provided	Not provided	82.6	≥2	Agar dilution
Jiang, 2017^90^	China	2014–15	126	STI clinic in hospital	Both, % not provided	Not provided	Not	100% urogenital	81.7	≥2	Agar dilution
Trecker, 2014^25^	China	2004–05, 2008–11	384	STI clinic in hospital	87.5	Not provided	14–83 (median 37)	Not provided	54.7	≥2	Agar dilution
Guoming, 2000^93^	China	1998–99	91	STI clinic	89.8	Not provided	Not provided	100% urogenital	49.5	≥2	Agar dilution
Lahra, 2022^36^	Australia	2021	3776	National surveillance	78.1	Not provided	Not provided	61.4% urogenital, 20.4% anorectal, 15.8% oropharynx. No site-specific MIC data	41.0	≥2	Agar dilution
Yu, 2017^88^	China	2012	244	STI clinic	92.2	Not provided	19–60	100% urogenital	32.4	≥2	Agar dilution
Lee, 2018^42^	New Zealand	2014–15	398	Population survey	81	Not provided	Not provided	Male: 64.6% urogenital, 16.8% anorectal, 13.7% penile, 2.8% oropharynx. Female: 90% cervical or vaginal. No site-specific MIC data	26.6	≥2	Agar dilution
Lee, 2016^40^	Korea	2010–11	70	Urology clinics	Not provided	Not provided	Not provided	Not provided	24.3	≥2	Agar dilution
Dewi, 2004^39^	Japan	1999–2001	131	Urology clinics	100	Not provided	Not provided	100% urogenital	17.6	≥2	Agar dilution
South Asia (*n* = 3; 168 isolates), median resistance 38.9% (range 12.0%–51%)											
Mal, 2016^43^	Pakistan	2012–14	100	Surveillance	Not provided	Not provided	Not provided	99% urogenital	51.0	≥2	Agar dilution
Sood, 2013^29^	India	2009–11	18	STI clinic in hospital	94.4	Not provided	15–50	100% urogenital	38.9	≥2	MIC gradient strip test (bioMérieux)
Ray, 2000^55^	India	1999	50	STI clinic in hospital	100	Not provided	Not provided	100% urogenital	12.0	≥2	MIC gradient strip test (AB BIODISK)
Europe and Central Asia (*n* = 20; 10 814 isolates), median resistance 50.5% (range 16.9%–100%)											
Chisholm, 2011^70^	England, Wales	2005–08	96	Surveillance	88	Yes (66% MSM)	17–67 (mean 31)	Not provided	100.0	≥2	Agar dilution, MIC gradient strip test (Bio-Stat)
Lucente, 2024^66^	Italy	2012–23	436	Infectious disease unit	99.7	Yes (99.1% MSM)	36.2 (IQR 30.6–43.7)	23.9% urogenital, 69% anorectal, 3.1% oropharynx. No site-specific MIC data	90.8	>0.5	MIC gradient strip test (bioMérieux)
Endimiani, 2014^27^	Switzerland	2009–12	34	Not provided	88.2	Not provided	21–56	94.1% urogenital, 2.9% oropharyngeal. No site-specific MIC data	88.2	>1	MIC gradient strip test (bioMérieux)
Brunner, 2014^53^	Hungary	2013	222	STI clinic	87	Yes (19% MSM)	14–76 (mean 31.7)	83.2% urogenital, 8.8% oropharyngeal, 8% anorectal. No site-specific MIC data	85.5	>1	MIC gradient strip test (Liofilchem)
UKHSA, 2022^72^	England, Wales	2021	1449	Surveillance of sexual health services	86.2	Yes (71.6% GBMSM)	15–82	53% urogenital (43.5 male and 9.5% female), 24.2% anorectal, 20.3% oropharynx. No site-specific MIC data	75.2	>1	Agar dilution
Brunner, 2016^54^	Hungary	2014	192	STI clinic	85	Not provided	Median 32 (male) and 26 (female)	Urogenital (female 65.5%–68.9%), anorectal (20.2%/44.8% male/female) and oropharynx (17.9%/24.1% male/female). No site-specific MIC data	70.0	>1	MIC gradient strip test (Liofilchem)
Unemo, 2024^34^	19 countries^d^	2022	4787	Surveillance	Not provided	Not provided	Not provided	Not provided	(a) 63.4 (b) 38.6	(a) >0.5 (b) >1	MIC gradient strip test (bioMérieux), Agar dilution
Regnath, 2015^52^	Germany	2010–15	266	Surveillance	75.6	Not provided	16–76 (median 34)	Not provided	55.6	>1	MIC gradient strip test (AB BIODISK)
Borovskaya, 2007^76^	Russia	2005–06	129	Not provided	Not provided	Not provided	Not provided	Not provided	54.0	≥2	Agar dilution
Młynarczyk-Bonikowska, 2016^44^	Poland	2012–13	65	STI clinic	86.2	Not provided	Not provided	96.9% urogenital (83.1% male, 13.8% female), 3.1% oropharynx. No site-specific MIC data	50.8	≥2	MIC gradient strip test (bioMérieux)
Carannante, 2014^64^	Italy	2009	114	Universities and STI clinics	90	Not provided	Median 33.2 male, 30.2 female	81.5% urogenital,11% anorectal, 1.2% oropharyngeal No site-specific MIC data	50.5	>1	MIC gradient strip test (bioMérieux)
Pitt, 2019^71^	England, Wales	2017	1268	Surveillance	Both (mostly men but % not stated)	Not provided	Not provided	Not provided	48.5	>1	Agar dilution
Ilina, 2008^75^	Russia	2004–05	464	STI clinics	Not provided	Not provided	Not provided	Not provided	38.4	≥2	Agar dilution
Enders, 2006^51^	Germany	2004–05	65	Specialist private clinics	85.5	Not provided	17–56 (mean 34)	100% urogenital	29.2	>2	MIC gradient strip test (AB BIODISK)
Kubanov, 2019^73^	Russia	2016	268	Surveillance	81.3	Not provided	12–64 (median 29)	100% urogenital	25.0	≥2	Agar dilution
Starnino, 2008^65^	Italy	2003–05	289	STI clinic	96.5	Not provided	Not provided	Male urethra (94.8%)	22.5	≥2	Agar dilution, MIC gradient strip test (AB BIODISK)
Shaskolskiy, 2018^77^	Russia	2015–17	401	Surveillance	80.3	Not provided	12–60	100% urogenital	19.0	>1	Agar dilution
Farhi, 2009^38^	France	2005–07	115	STI clinic	95.6	Yes (73% MSM)	17–67 (median 30)	74.8% urogenital, 19.1% anorectal, pharynx + anorectal (7.8%), anorectal + urethral + pharyngeal (1.7%). No site-specific MIC data	17.4	Per CA-SFM, >1	Agar dilution
Calado, 2019^45^	Portugal	2013–15	30	STI clinic	100	Yes (100% MSM)	Not provided	83.3% rectal, 16.7% urethral. No site-specific MIC data	17.0	>1	MIC gradient strip test (Oxoid)
Kubanov, 2016^74^	Russia	2015	124	Surveillance	74.2	Not provided	Not provided	100% urogenital	16.9	>1	Agar dilution
Latin America and the Caribbean (*n* = 7; 2334 isolates), median resistance 61.6% (range 22.6%–100%)											
Vacchino, 2017^50^	Argentina	2014–15	40	Public hospital	Not provided	Not provided	Not provided	97.5% urogenital	100	≥2	Agar dilution method
Sosa, 2003^63^	Cuba	1995–98	91	Provincial health centres	Both	Not provided	Not provided	Urogenital, rectal and conjunctival—% not provided	83.5	≥2	Agar dilution
da Costa-Lourenço, 2018^26^	Brazil	2005–15	116	Public/private health facilities, private laboratories	86.6	Not provided	13–70	98.3% urogenital, 1.7% anorectal. No site-specific MIC data	65.5	≥2	Agar dilution
Bazzo, 2018^61^	Brazil	2015–16	550	Surveillance	100	Not provided	>18	100% urogenital	61.6	≥2	Agar dilution Method
Llanes, 2003^62^	Cuba	1995–99	120	Surveillance	Both, % not provided	Not provided	Not provided	100% urogenital	54.2	≥2	Agar dilution
Costa, 2013^60^	Brazil	2011–12	201	STI clinic	95	Yes (21% MSM)	14–61	100% urogenital	32.3	≥2	MIC gradient strip test (bioMérieux)
Thakur, 2017^37^	Argentina, Bolivia, Chile, Colombia, Cuba, Uruguay, Venezuela	2011	1216	Surveillance	Not provided	Not provided	Not provided	Not provided	22.6	≥2	Agar dilution, MIC gradient strip test (bioMérieux) or disc diffusion (country dependent)
Middle East and North Africa (*n* = 1; 46 isolates)											
Al-Maslamani, 2022^46^	Qatar	2020	46	Public/private clinics	96.5	Not provided	13–77 (median 24)	100% urogenital	41.3	≥2	MIC gradient strip test (bioMérieux)
North America (*n* = 10; 59 246 isolates), median resistance 26.5% (range 12.0%–51%)											
Eyre, 2017^33^	UK, USA, Canada	UK (July 2004–14), USA (2009–10), Canada (1989–2014)	681	Surveillance	Not provided	Not provided	Not provided	Not provided	77.7	>1	Agar dilution
Sawatzky, 2023^67^	Canada	2021	3439	Surveillance	84.1	Not provided	24.9% (<26); 53.7% (26–40)	55.6% urogenital, 19.9% anorectal, 20.6% oropharynx. No site-specific MIC data	65.9	≥2	Agar dilution
Ma, 2023^69^	Canada	2017–20	2394	Public health facilities	69.1	Not provided	20–39	43.7 urogenital, 22.3% anorectal, 20.4% oropharynx. No site-specific MIC data	65.8	≥2	Agar dilution
Quilter, 2021^24^	USA	2018–19	6576	Surveillance STI clinics	100	100% MSM	72% under 35	60.4% urogenital, 23.6 anorectal, 16.0% oropharyngeal. Does provide site-specific MIC data	34.5	≥2	Agar dilution
Reimche, 2023^83^	USA	2019	1710	Surveillance	100	Yes (27% MSM)	24% (25–29)	100% urogenital	27.6	≥2	Agar dilution
Kirkcaldy, 2016^85^	USA	2014	5093	Surveillance, STI clinic	100	Yes (37.1% were MSM)	median 28	100% urogenital	25.3	≥2	Agar dilution
Kidd, 2015^84^	USA	2010–12	478	STI clinics	88.3	Yes (35.6% MSM)	15–60	100% urogenital	24.0	≥2	Agar dilution
Kirkcaldy, 2013^35^	USA	2005–10	34 600	Surveillance	100	Yes (23.5% MSM)	58.9% >24	100% urogenital	18.9	≥2	Agar dilution
Ng, 2003^68^	Canada	1999	4025	STI clinics	Both, % not provided	Not provided	Not provided	All sites (rectal, pharyngeal, urethral, vaginal, cervical)	10.9	≥2	Agar dilution
Herchline, 2010^86^	USA	2006–08	250	STI clinics	Both, % not provided	Not provided	Not provided	Not provided	4.0	≥2	MIC gradient strip test (AB BIODISK)

CA-SFM, Comité de l’antibiogramme de la Société Française de Microbiologie; GBMSM, gay, bisexual MSM; NG-MAST, *N. gonorrhoeae* multiantigen sequence typing.

^a^World Bank regions.

^b^Both means both sexes included but no % by sex provided.

^c^EUCAST breakpoint before 2023 (www.eucast.org/clinical_breakpoints), CLSI (https://clsi.org/).

^d^Austria, Belgium, Bulgaria, Czechia, Estonia, France, Germany, Greece, Hungary, Iceland, Malta, The Netherlands, Norway, Poland, Portugal, Slovakia, Slovenia, Spain, Sweden.

### Resistance over time

Among the 80 645 isolates studied, 91.4% were collected in the most recent period (2010–23). Largest increases in tetracycline resistance between the two reporting periods (2010–23 versus 1996–2009) were seen in South Asia and North America, with a 3.8- and 4.1-fold relative increase, respectively (Table 2). All other regions reported similar resistance between the two periods, except Europe and Central Asia, showing a 1.4-fold increase.

**Table 2.** Tetracycline resistance in *N. gonorrhoeae* by World Bank region over time

World Bank region	1996–2009 (% Resistance)		2010–23 (% Resistance)		Overall (1996–2023) (% Resistance)		Relative resistance increases (2010–23 versus 1996–2009)
	Median	Range	Median	Range	Median	Range	
East Asia and Pacific (*n* = 16; 6304 isolates)	82.1 (*n* = 4; 659 isolates)	17.6–100	82.1 (*n* = 12; 5645 isolates)	24.3–98.7	82.1	17.6–100	1.0
Sub-Saharan Africa (*n* = 10; 1733 isolates)	75.1 (*n* = 3; 450 isolates)	54.0–77.0	87.0 (*n* = 7; 1283 isolates)	44.0–100	81.6	44.0–100	1.2
Latin America and Caribbean (*n* = 7; 2344 isolates)	63.6 (*n* = 2; 211 isolates)	54.2–83.5	61.6 (*n* = 5; 2123 isolates)	22.6–100	61.6	22.6–100	0.97
Europe and Central Asia (*n* = 20; 10 814 isolates)	38.4 (*n* = 7; 1272 isolates)	17.4–100.0	55.6 (*n* = 13; 9542 isolates)	16.9–90.8	50.7	16.9–100	1.4
Middle East and North Africa (*n* = 1; 46 isolates)			41.3 (*n* = 1; 46 isolates)		41.3	—	—
South Asia (*n* = 3; 168 isolates)	12.0 (*n* = 1; 50 isolates)	—	45.0 (*n* = 2; 118 isolates)	38.9–51.0	38.9	12.0–51.0	3.8
North America (*n* = 10; 59 246 isolates)	7.5 (*n* = 2; 4275 isolates)	4.0–10.9	31.1 (*n* = 8; 54,971 isolates)	18.9–77.7	26.5	4.0–77.7	4.1

## Discussion

The present systematic review including 67 studies, data from 51 countries and 80 645 isolates between 1996 to 2023 found the median *N. gonorrhoeae* tetracycline resistance to be 54.2% (range: 4%–100%)—higher in East Asia and Pacific (82.1%) and sub-Saharan Africa (81.6%) and lowest in North America (26.5%). A nearly 4-fold increase in tetracycline resistance was seen in South Asia and North America in 2010–23 versus 1996–2009.

Our findings support other findings within sub-Saharan Africa and Europe, which reported 100% tetracycline resistance found in *N. gonorrhoeae* isolates from a doxyPEP trial in Kenyan women^12^ [due to the *tet*(M) gene] and from MSM from France.^8^ Our East Asia and Pacific (82.1%) estimates are similar to those in a recent surveillance report of *N. gonorrhoeae* tetracycline resistance (not included in our analysis as it was published outside of our search period) of 92.2% and 80.6%^95^ based on MIC > 0.5 mg/L and MIC > 1.0 mg/L, respectively. Including these data in our analysis would have resulted in similar results—overall resistance of 54.5% (versus 54.2%) and East Asia and Pacific 82.6% (versus 82.1%). The 4-fold relative increase in resistance in North America between the two reporting periods is also of concern. While North America’s resistance was the lowest in our analysis, the impacts on *N. gonorrhoeae* incidence have been variable, ranging from a 1.8% *increase* per month in *N. gonorrhoeae* notifications in the 1 year post-doxyPEP implementation phase,^11^ 12% reduction using electronic health data^96^ (with no impact on oropharyngeal infections) or a 55% reduction using a clinical trial.^6^ Similarly, in Kings County (USA) high-level tetracycline resistance was reported in the last year after the introduction of doxyPEP, with resistance being associated with those taking more than three doses of doxyPEP.^97^ Therefore, doxyPEP may be unreliable to prevent incident gonococcal infections. While tetracyclines are not used to treat *N. gonorrhoeae* globally, our finding suggests doxyPEP would have a very limited role in *N. gonorrhoeae* control when used to reduce incidence of other STIs such as chlamydia and syphilis, and as such, doxyPEP policies should make clear statements to prescribers and users regarding this. With changes in the recommended treatment of chlamydia from azithromycin to doxycycline to improve its efficacy against anorectal infections,^98^ this increase in overall doxycycline consumption may also impact tetracycline resistance in *N. gonorrhoeae*.

As the US CDC implements doxyPEP, it will remain critical that their GISP and particularly their enhanced Project (eGISP) examine resistance at extragenital sites and by sex, to be robust given the difference in MICs seen between sexes and infection sites.^99,100^ This is especially pertinent for the oropharyngeal site, where treatment failures are more frequent,^101^ and among MSM, where transmission of AMR *N. gonorrhoeae* strains between partners remains a major factor in gonococcal AMR.^102^ However, it would be genital samples from women that would be the most important to monitor as *cis*-women would bear the greatest harm from infections compared with MSM due to the negative reproductive sequelae of gonorrhoea. Additionally, while the long-term impacts on *N. gonorrhoeae* AMR from doxyPEP remain unclear, genomic studies of *N. gonorrhoeae* isolates from the USA^103^ and others^104,105^ have reported that if all tetracycline-resistant *N. gonorrhoeae* lineages are selected, because these lineages are most frequently also resistant to additional antimicrobials, these other antibiotic classes, including first-line treatments such as cephalosporins, may be threatened. US surveillance data reported that gonococcal isolates from MSM were significantly more likely than men who have sex with women (adjusted OR > 2) to exhibit resistance to antibiotics including cephalosporins and tetracycline.^35^ As we see initial increases in *N. gonorrhoeae* notifications from San Francisco post-doxyPEP implementation after seeing previous declines,^11^ we await any genomic analysis of those isolates as part of the recommended monitoring by the CDC doxyPEP guidelines.

The prevalence of *N. gonorrhoeae* is highest (up to 10%) among MSM at the oropharynx or anorectum,^106^ and *N. gonorrhoeae* recovered from oropharyngeal infections can have higher MICs than those from urogenital infections.^107^ However, in this review, only 18% (12/67) of studies were from the anorectum or oropharyngeal sites, with only 11 studies (16%) providing tetracycline resistance data specifically from MSM, who constituted between 19% and 100% of study isolates. As *N. gonorrhoeae* incidence continues to rise annually around the world^3–5^ it is essential that *N. gonorrhoeae* AMR surveillance systems support AMR monitoring during doxyPEP implementation, as recommended by these policies. However, surveillance systems are not universally optimal. Medland *et al.*^108^ reported that up to September 2020, among 32 *N. gonorrhoeae* national surveillance systems, 20 (62.5%) monitored for tetracycline resistance (excluding many in European countries) and only 13 (41%) collected non-anogenital samples from men. The study reported major gaps in coverage and representativeness of these systems to monitor and respond to *N. gonorrhoeae* AMR, which poses a global risk due to the interconnectedness of the world through travel. This was particularly striking in low- and middle-income countries with higher burdens of gonorrhoea and where small numbers of *N. gonorrhoeae* isolates were collected for AMR testing. This will need to be addressed as recent modelling of USA data found that lower sample numbers can delay detection of significant increases in tetracycline resistance prospectively.^109^ Countries implementing doxyPEP should ensure AMR systems are in place to monitor *N. gonorrhoeae* AMR and, probably more importantly, for non-STIs such as *S. aureus*^97^ as resistance in these infections can be fatal, unlike for *N. gonorrhoeae* infections. Having existing systems to monitor *N. gonorrhoeae* AMR does not necessarily mean that they are ready without significant enhancements to the system.^110^

Lastly, data for women were limited. While doxyPEP is not recommended for *cis*-women, STI infections pose significant harm (i.e. reproductive) in women compared with MSM. Our review only found one study that exclusively studied *N. gonorrhoeae* isolates from women^56^ (85 isolates) or a substantial proportion of women (48%–56% of total isolates, 40 isolates across these studies)^41,59^ and reported tetracycline resistances between 87% and 100%. Risk of *N. gonorrhoeae* AMR can be significant in women, with an Australian study of 7588 *N. gonorrhoeae* isolates (2007–18) reporting the odds of MDR *N. gonorrhoeae* being twice as high in women compared with MSM,^111^ and UK surveillance of 10 275 isolates reporting that pharyngeal *N. gonorrhoeae* infections from women (and MSM) had reduced susceptibility to ceftriaxone compared with other infection sites.^107^

The present review had some limitations. Many studies provided aggregated, averaged tetracycline resistance data from men, women and different sites of infection. This review then calculated a median estimate from these data. Therefore, calculating a median from other summary estimates may lack precision. Fifteen percent (10/67) of studies had 50 or fewer isolates, including Qatar—the only country representing the Middle East and North Africa region. The diversity of countries and years the studies were undertaken, differences in sampling, tetracycline resistance breakpoints used and methods used to assess tetracycline susceptibility make comparisons between studies difficult. However, all but one study^66^ used identical tetracycline resistance breakpoints, i.e. from EUCAST (>1 mg/L before 2023) or US CLSI (≥2 mg/L). Some tetracycline resistance data were not recent, with only 18% (12/67) of studies from 2019–23, and we assumed that MICs did not decrease over time to the present time for this review. However, 91% of isolates in this review were collected between 2010 and 2023. It is possible, but unlikely, that *N. gonorrhoeae* resistance might have decreased over time, but our analysis found resistance to be the similar or increased between the two reporting periods, and a recent report showed increasing resistance to tetracycline and ciprofloxacin over time.^112^ It is possible that some relevant studies may have been missed but this would not significantly change readers’ interpretations for countries where data are presented, especially those with smaller sample sizes. Lastly, data from several World Bank regions were represented by only a few countries and single sites within each country; this may have been represented by data from a certain region and not nationally. For example, the Middle East/North Africa region only has data from one country (Qatar, 46 isolates) and South Asia from one country (50 isolates) between 1996 and 2009. Therefore, resistance data may not be generalizable to that country or region. Similarly, country-level data may have included travellers rather than local residents in the country of study but travellers are often significant contributors to *N. gonorrhoeae* AMR transmission.^36,85,111^

### Conclusions

This systematic review reveals that doxyPEP is unlikely to profoundly reduce *N. gonorrhoeae* incidence globally. Additionally, the rates of tetracycline/doxycycline resistance appear to be trending upwards, particularly in the regions that had lower rates of tetracycline/doxycycline resistance in 1996–2009. Finally, our findings strongly emphasize the need to optimize *N. gonorrhoeae* surveillance systems, including the collection of more data from women, MSM, bisexual men and from the oropharynx.

## Supplementary Material

dlaf120_Supplementary_Data

## Data Availability

The authors confirm that the data supporting the findings of this study are available within the article.

## Supplementary data

Table S1 and S2 are available as Supplementary data at *JAC-AMR* Online.
